# The Relation of Serum Vitamin C Concentrations with Alzheimer’s Disease Mortality in a National Cohort of Community-Dwelling Elderly Adults

**DOI:** 10.3390/nu16111672

**Published:** 2024-05-29

**Authors:** Duke Appiah, Elyvine Ingabire-Gasana, Linda Appiah, Jeanne Yang

**Affiliations:** 1 Department of Public Health, Texas Tech University Health Sciences Center, Lubbock, TX 79430, USA; 2 Department of Nutritional Sciences, Texas Tech University, Lubbock, TX 79409, USA; 3 College of Education, Texas Tech University, Lubbock, TX 79409, USA; 4 School of Medicine, Texas Tech University Health Sciences Center, Lubbock, TX 79430, USA

**Keywords:** Alzheimer’s disease, vitamin C, mortality, epidemiology

## Abstract

The relation of vitamin C with Alzheimer’s disease (AD) is equivocal. The aim of this study was to assess the relation of serum vitamin C levels with AD-related mortality, and to evaluate the threshold beyond which the potential benefits of higher serum concentrations of vitamin C for AD mortality ceases. The cohort consisted of 4504 adults aged ≥60 years enrolled in the National Health and Nutrition Examination Survey who had serum measures of vitamin C and no cognitive impairment at baseline (1988–1994) and were followed-up for mortality until 2019. Vitamin C was assayed from fasting blood samples using isocratic high-performance liquid chromatography. At baseline, the mean age of participants was 70 years, with 42.7% being men. At the end of follow-up (median: 15 years), the AD mortality rate was 2.4 per 1000 person-years. In the Cox regression models, compared to participants in the lowest tertile of serum vitamin C (<0.56 mg/dL), those in the highest tertile (>0.98 mg/dL) had a lower risk of AD mortality (hazard ratio: 0.44, 95% confidence intervals: 0.25–0.77) after adjusting for sociodemographic factors, behavior/lifestyle factors, prevalent health conditions, and dietary vitamin C intake. In dose–response analysis using restricted cubic splines, vitamin C concentrations beyond 2.3 mg/dL were associated with the elevated risk of AD-related mortality. The findings from this national sample of community-dwelling elderly adults suggest that higher levels of serum vitamin C are associated with slower AD disease progression, although levels beyond the normal reference values were associated with a higher risk of AD mortality.

## 1. Introduction

Alzheimer’s disease (AD) is an irreversible heterogenous neurodegenerative disease that contributes substantially to morbidity, reduced quality of life, and premature mortality [1]. This incurable disease, which affects 6 million Americans and is associated with an estimated $321 billion in healthcare costs, is characterized by memory loss, impaired cognition, and limitations in performing activities of daily living [2]. Over the past two decades, AD-related mortality has increased by more than 146.2% [1]. This multifactorial disease, which occurs largely due to the complex interplay of genetic susceptibility and behavioral and environmental factors over the lifespan, has a long prodromal period, suggesting that some early interventions may delay the onset of AD [1]. Accordingly, about one in three cases of AD have been attributed to underlying modifiable risk factors [1,3].

Vitamin C or ascorbic acid is an antioxidant that has been reported to play important roles in the prevention and treatment of several acute and chronic conditions, including the common cold, diabetes, and cancers [4,5,6,7]. Vitamin C, being a redox catalyst, is known to be a scavenger of free radicals during cellular metabolic processes [2]. In humans, it has been observed that the highest concentration of vitamin C is found in the brain (cerebral cortex, hippocampus, and amygdala) as well as the cerebrospinal fluid, where the levels are reported to be higher than vitamin C levels in the plasma [6]. In the brains of some patients with AD, the formation and deposition of neurofibrillary tangles and Aβ plaques, which is probably associated with oxidative stress, has been reported [6]. The increased generation of free radicals in the presence of neurodegenerative disorders has cultivated great interest in understanding the role of vitamin C in delaying the onset of AD [8]. 

Accordingly, vitamin C is reported to play significant roles in neurotransmission, and may potentially mediate neural oxidative imbalance and inflammation—three factors that have been implicated in the pathogenesis of AD [2]. In animal studies, vitamin C was observed to reduce oxidative stress that often results in amyloid-β-induced apoptosis and death in neurons [9]. In settings of reduced vitamin C, as in a vitamin C deficiency state, accelerated amyloid pathogenesis and deposition [10] as well as reduced levels of dopamine, which is very susceptible to oxidation and plays an important role in cognition [11], have been observed. Deficits in dopaminergic neurotransmission have been reported to lead to the progression of AD [12]. Vitamin C is also known to reduce neuroinflammation by modulating microglial polarization and astrocyte activation [13]. To add to that, vitamin C, in combination with other vitamins and minerals, is reported to slow the progression of AD in rats by increasing brain monoamines, superoxide dismutase, total antioxidant capacity, and brain-derived neurotrophic factor as well as mitigating brain acetylcholine esterase, β-amyloid, tau protein, β-secretase, malondialdehyde, tumor necrosis factor-alpha, Interleukin 1β, and DNA fragmentation [13,14].

However, the evidence for the potential beneficial effect of dietary vitamin C, vitamin C supplementation, or serum concentration of vitamin C, a good indicator of dietary intake of fruits and vegetables, on the incidence or mortality due to AD, remains equivocal [6,15,16,17,18,19]. Some of the reasons attributed to the divergent results include inaccuracies in the classification of participants according to vitamin C status, or the inadequate assessment of daily dietary vitamin C intake, which is sometimes obtained from food diaries [20]. Very few of the studies evaluating the role of blood levels of vitamin C and AD were conducted in ethnically diverse populations or provided evidence from prospective studies. Additionally, the threshold beyond which the potential benefits of higher circulating levels of vitamin C ceases for reducing the risk of AD is largely unknown.

The aim of this study was twofold: first, to evaluate the association of serum concentrations of vitamin C with AD-related mortality in a cohort of community-dwelling elderly adults in the United States; second, to evaluate the highest threshold beyond which the potential benefits of higher serum concentrations of vitamin C for the reduced risk of AD-related mortality ceases.

## 2. Materials and Methods

### 2.1. Study Population

Data on the community-dwelling elderly adults were obtained from the third National Health and Nutrition Examination Survey (NHANES III, 1988–1994). This survey used a stratified, multistage, probability cluster sampling to obtain a nationally representative sample of the population of the United States who were not institutionalized [21]. Of the 5302 community-dwelling elderly adults aged 60 years and older who participated in the NHANES medical exam, we excluded 260 individuals with cognitive impairment, and 402 participants with missing data for serum vitamin C concentrations. Furthermore, 136 individuals who died within a year of their examination were excluded. This was carried out because such individuals may have had a high risk of mortality, and therefore might have changed their behavior or lifestyle pertaining to their diet and/or dietary supplement intake, thus influencing their serum vitamin C concentrations. The remaining 4504 participants after all the exclusions were included in the current study. Approval from an institutional review board was not required for the current study since NHANES data are de-identified and publicly available. 

### 2.2. Vitamin C

During the morning examination session at the mobile centers, blood samples were obtained using standard venipuncture procedures from all participants who were asked to fast overnight. One part serum was mixed with four parts 6% metaphosphoric acid to acidify the serum and stabilize it [22]. This was then frozen at −70 °C and shipped to the Centers for Disease Control and Prevention laboratory for further processing and analysis. The specimen was later thawed at room temperature, and serum vitamin C was measured using isocratic high-performance liquid chromatography with electrochemical detection at 650 mV (Waters Chromatography Division of Millipore Corporation, Marlboro, MA, USA) [22]. The coefficient of variation for the vitamin C assay in NHANES III averaged 5.8% [22]. In NHANES III (1988–1994), vitamin C was measured only once. Details of the laboratory procedures for NHANES III are reported elsewhere [22]. The current normal range for vitamin C in the U.S. population is reported to be 0.11–2.04 mg/dL [23].

### 2.3. AD Mortality

The International Classification of Diseases tenth version (ICD-10) code G30 was used to define AD mortality occurring after the NHANES medical examination through to the end of 2019. The National Center for Health Statistics matched the information of adult participants with death certificate information from the National Death Index using a probabilistic matching methodology to ascertain vital status as well as document the underlying cause of death for participants. 

### 2.4. Measures

Several potential factors related to serum vitamin C levels were ascertained in NHANES III. These include sociodemographic factors, namely age at baseline, sex, race, ethnicity, years of education, marital status, and household income, as well as behavior and lifestyle factors such as smoking status, alcohol intake, and physical activity. All these factors were self-reported by participants. A poverty–income ratio of less than 1.3 was used to define low-income status. Information on diets that participants consumed in the past 24 h was gathered via dietary recall interviews (recalls were limited to weekdays because of the examination schedules), with diet quality evaluated using the Healthy Eating Index. Details of the assessment of dietary intake in NHANES III are provided elsewhere [24]. Briefly, dietary intake was evaluated for all participants at the Mobile Examination Center by trained dietary interviewers, fluent in English and Spanish, using an automated computer-based interview and coding system [25,26]. Data collection took place on all days of the week. Participants were asked about the recall of all foods and beverages eaten in the past 24 h. Additionally, they were further asked if the intake of the reported foods was their usual intake for that day [26]. Recall aids including charts, abstract food models, and measuring cups were used to aid in quantifying the portion sizes of food consumed by the participants [25]. Whenever needed, interviewers probed for the consumption of some often-forgotten foods such as condiments, fast foods, and alcoholic beverages [25]. The nutritional contents of the reported foods were derived using the U.S. Department of Agriculture Survey Nutrient Database [24,25,26].

Participants were also asked about vitamin or mineral supplements taken in the previous month. Vitamin C supplement use was obtained by the review of nutritional labels of medications taken over the past month provided by participants. 

Trained interviewers measured participants’ height and weight using standardized procedures. We calculated body mass index by dividing participants’ weight in kilograms by the square of their height in meters. Abdominal obesity was defined as waist circumference greater than 103 cm for men and 88 cm for women. Physical activity was measured by asking participants if, over the past month, they have participated in leisure time activities like walking a mile without stopping, jogging or running, bicycle or exercise bike riding, lifting weights, swimming, aerobics, dancing, calisthenics, and working in a garden or yard. The frequency of these and other activities were multiplied by their corresponding metabolic equivalent intensity scores and divided into tertiles, with the lowest tertile considered to be sedentary. Total cholesterol was measured from fasting blood samples. With regard to prevalent medical conditions, hypertension was defined as elevated systolic (>130 mm Hg) or diastolic (>80 mm Hg) blood pressure, or current use of antihypertensive medications. Diabetes was defined as either a self-report of physician diagnosis of diabetes, fasting blood glucose levels ≥126 mg/dL, oral glucose tolerance test ≥200 mg/dL, or the self-reported use of diabetes medications. Cancer and cardiovascular disease were all self-reported. 

In NHANES III, cognitive function was assessed using six neuropsychological tests focusing on orientation, attention, and verbal memory (both immediate and delayed). These tests were administered in either English or Spanish. Orientation in time and place, as well as verbal memory, were assessed using object and story recall tasks based on several metrics, including the Mini-Mental State Examination Scale, Weschler Adult Intelligence Scale, and the East Boston Memory Test [27]. A global cognitive function score was estimated by adding up the standardized scores for each of the six neuropsychological tests. Similar to other studies [27,28], cognitive impairment was defined as a score in the lowest 10% of the distribution of the global cognitive function score.

### 2.5. Statistical Analysis

Due to the skewed distribution of serum vitamin C, it is categorized into tertiles. Participants’ characteristics at baseline were presented according to tertiles of serum vitamin C, with the comparison of categorical variables made using chi-squared tests, while Analysis of Variance was used for comparisons of continuous variables. In time-to-event analysis, the cumulative incidence of deaths due to AD was estimated using the Fine and Gray method [29]. All other deaths not due to AD were considered as competing risk events. We calculated hazard ratios (HR) and their 95% confidence intervals (CIs) using Cox regression models. Progressive adjustments were made for factors reported in the literature to influence serum vitamin C levels or AD. Model 1 adjusted for sociodemographic factors (age, sex, race/ethnicity, education, poverty–income ratio, and marital status). Model 2 further adjusted for behavior/lifestyle and metabolic factors (systolic blood pressure, smoking status, body mass index, abdominal obesity, physical activity, and total cholesterol). Model 3 additionally adjusted for prevalent chronic health conditions at baseline (diabetes, cardiovascular disease, and cancer). Finally, model 4 adjusted for dietary intake of vitamin C. The complex survey design and sampling weights were incorporated in the analysis. We tested and confirmed the proportional hazard assumption using interaction terms between tertiles of serum vitamin C and the log of follow-up time. Two-way interactions between serum vitamin C and sociodemographic and behavior/lifestyle factors were tested and reported whenever statistically significant. We performed additional analyses using established cutoff points for blood concentrations of vitamin C [20,30]. Similar to other studies from NHANES III [31,32], we defined serum levels of vitamin C < 11 µmol/L (0.2 mg/dL) as deficient, 11 to 28 µmol/L (0.2 to 0.5 mg/dL) as low or marginally deficient, and >29 µmol/L (0.5 mg/dL) as adequate or optimal. Due to small samples limiting statistical power, the deficient and low groups were combined and classified as inadequate. Restricted cubic splines were used to evaluate the highest threshold beyond which the potential benefits of higher concentrations of serum vitamin C for reduced risk of AD-related mortality ceases [33]. Statistical significance was assessed using a *p* value < 0.05, with the SAS software version 9.4 (SAS Institute) used for the analyses.

## 3. Results

The mean age of the 4504 participants (weighted to 33.5 million adults in the United States) at baseline was 70.0 years, with 42.7% being men. About 86% of participants reported non-Hispanic White race and ethnicity while 7.4%, 2.2%, and 4.9% reported non-Hispanic Black, Mexican American, and other racial or ethnic groups, respectively. Characteristics of participants according to tertiles of serum vitamin C concentrations are reported in Table 1. On the one hand, the proportion of elderly adults who were non-Hispanic White, had some college degree or higher, and were taking vitamin C supplements, as well as baseline age, dietary vitamin C intake, and diet quality scores increased with higher serum concentrations of vitamin C. On the other hand, the proportion of men, low-income status, sedentary lifestyle, diabetes, and cancer decreased with higher levels of serum vitamin C. The prevalence of vitamin C deficiency was 8.9%, while 14.9% of participants were observed to have low serum vitamin C levels.

**Figure 1:**
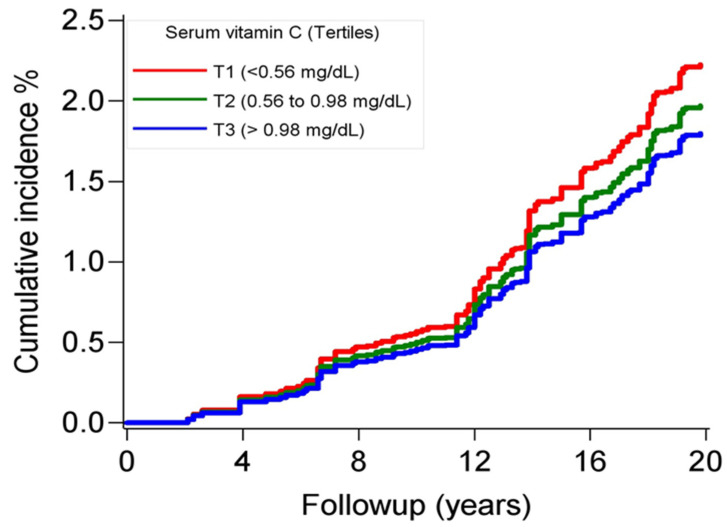
Cumulative incidence curves for deaths due to Alzheimer’s disease according to tertiles of serum vitamin C concentrations, NHANES, 1988–2019. The *p* value of the Gray’s test for equality of the curves was <0.01.

During a median follow-up of 15 years, 153 deaths due to AD were documented, resulting in a mortality of 2.4 per 1000 person-years. AD mortality was highest among participants in the lowest tertile of serum vitamin C (Figure 1). In the Cox regression models, adjusting for sociodemographic factors, behavior/lifestyle factors, metabolic factors, and prevalent health conditions, compared to the lowest tertile of serum vitamin C, those in the highest tertile had a lower risk for AD mortality (HR: 0.50, 95% CI: 0.30–0.81) (Table 2). Further adjustment for dietary vitamin C intake strengthened this association (HR: 0.44, 95% CI: 0.25–0.77). No significant interactions were found between serum vitamin C and sociodemographic or behavior/lifestyle factors on the risk of AD mortality.

Compared to participants with inadequate concentrations of serum vitamin C, thus deficient and low status, those with adequate concentrations had a 41% reduced risk for AD mortality after adjusting for sociodemographic factors, behavior/lifestyle factors, metabolic factors, and prevalent health conditions (HR: 0.59, 95% CI: 0.36–0.97, *p* = 0.039). However, the risk for AD mortality reduced to 46% when an additional adjustment was made for dietary intake of vitamin C (HR: 0.54, 95% CI: 0.29–0.99, *p* = 0.049). 

**Figure 2:**
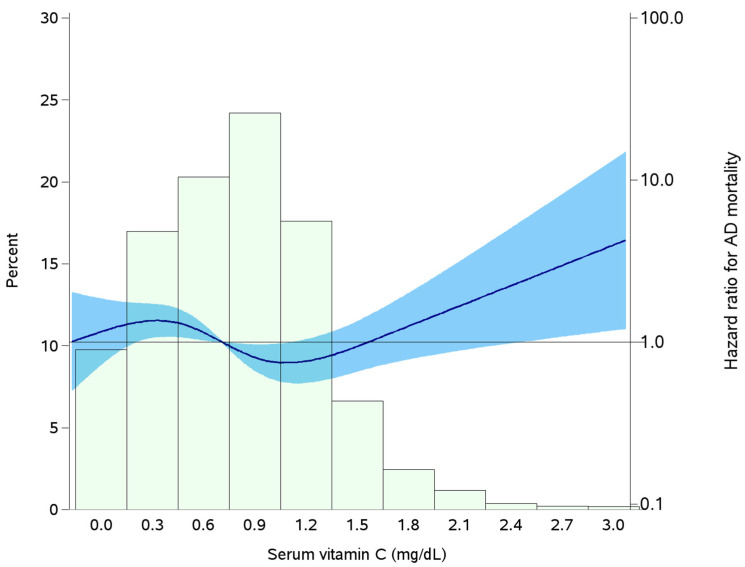
Adjusted restricted cubic splines for the relation of serum vitamin C levels and Alzheimer’s disease mortality. Model adjusted for age, sex, race/ethnicity, education, poverty–income ratio, marital status, systolic blood pressure, smoking status, body mass index, physical activity, total cholesterol, and prevalent chronic conditions at baseline (diabetes, cardiovascular disease, and cancer). Splines, thus the shaded (light blue) regions bounded by the lines, represent hazard ratios and their 95% confidence intervals. The referent concentration of serum vitamin C was set at the median level (0.79 mg/dL). The bars (light green) represent a histogram of the percent of elderly adults included in the study at the various serum concentrations of vitamin C.

The results from the restricted cubic spline analysis supported a non-linear association between serum vitamin C and AD mortality (*p* = 0.0174). In this analysis, the concentrations of serum vitamin C which were associated with a lower risk for AD mortality were from 0.80 mg/dL to 1.09 mg/dL, with concentrations above 2.3 mg/dL associated with an elevated risk for AD mortality (HR: 2.48, 95% CI: 1.03–5.96) (Figure 2). Overall, only 1.0% of the population had serum vitamin C concentrations above 2.3 mg/dL, with the prevalence higher in participants taking vitamin C supplements (2.0% vs. 0.4%, *p* = 0.010).

## 4. Discussion

In this large national cohort of racially and ethnically diverse community-dwelling elderly adults in the United States, an inverse relationship was observed between serum vitamin C levels and AD mortality, independent of sociodemographic and behavior/lifestyle factors, metabolic factors, chronic health conditions, and dietary vitamin C intake. However, beyond serum vitamin C concentrations of 2.3 mg/dL, an elevated risk for AD mortality was observed.

Potential beneficial effects of vitamin C on the development of AD in humans have also been observed in some studies [6,34,35,36,37,38]. A cross-sectional study of 424 Cubans aged ≥65 years reported significantly lower blood levels of vitamin C among individuals with mild cognitive impairment or AD [35]. Similarly, two meta-analyses of cross-sectional and case-control studies [6,37] concluded that levels of vitamin C were lower among individuals with AD compared to healthy controls. The current study extends the findings of these studies as it provides stronger evidence for the inverse association of serum vitamin C levels and AD, as a prospective design with a long duration of follow-up was implemented with several confounding factors accounted for in the analysis. To add to that, a mendelian randomization by Chen et al. [17] based on 11 single-nucleotide polymorphisms reported that higher concentrations of vitamin C in the blood are casually associated with a decreased risk of AD. Similarly, studies on vitamin C supplementation [39,40] or intake of fruits and vegetables high in vitamin C [41,42,43] have largely also shown an inverse relationship between vitamin C and the incidence, progression, or alleviation of some of the symptoms associated with AD [6]. 

Conversely, other studies report a weak or no association between blood levels or vitamin C supplement use and AD [44,45,46,47]. In a small study of 50 Japanese adults with AD with an average age of 76 years, a weak correlation was found between plasma and lymphocyte vitamin C levels and the progression of cognitive dysfunction [45]. In the Honolulu–Asia Aging Study of Japanese American men living in Hawaii, although vitamin C supplements were reported to be protective against vascular dementia and improved cognitive function, an independent association was not observed between vitamin C supplement use and AD [44]. In the Washington Heights–Inwood Columbia Aging Project, neither dietary, supplemental, nor total intake of vitamin C showed any benefit in the development of AD [48]. In one of the few clinical trials conducted to date to investigate a potential link between vitamin C and AD, vitamin C supplements as well as other antioxidants did not influence cerebrospinal fluid biomarkers related to amyloid or tau pathology [49]. Interestingly, a mendelian randomization by Liu et al. [50], using the same cohort and same number of plasma vitamin C genetic variants as Chen et al. [17], came to different conclusions, with the former reporting that plasma vitamin C may not be causally related with AD.

The diverging results in the association of blood levels of vitamin C with AD may in part be explained by differences pertaining to study designs, duration of follow-up, limited sample sizes, the operationalization of vitamin C in regression models, the degree of adjustment for confounding factors including vitamin C supplement intake, and differences in ages of participants at the time of vitamin C assessment. For instance, relatively younger populations were enrolled in most prospective or randomized trials, compared to other study designs like case-control and cross-sectional studies [18]. It is worth noting that several of the previous studies on the association of vitamin C and AD, especially the mendelian randomization studies, were based on homogeneous populations that were often of European ancestry. Thus, these findings may be different among racial and ethnic minority groups who are known to have a higher risk of AD compared to non-Hispanic White populations [51].

The actual value of vitamin C that represents optimal health for chronic diseases is unknown [20]. Although the current upper limit for normal serum vitamin C levels in the U.S. population is 2.04 mg/dL [23], this does not necessarily translate to risk for diseases. Another significant finding from the current study is that extremely high levels of serum vitamin C concentrations beyond 2.3 mg/dL are associated with a higher risk of AD, suggesting this to be a threshold beyond which the potential benefit of serum vitamin C concentrations on the risk of AD ceases. Higher serum levels of vitamin C are often linked to higher dietary intake of a diet rich in vitamin C, or vitamin C supplement intake [20]. While the mechanism linking very high levels of vitamin C to AD progression is unknown, some reports speculate that high levels of vitamin C may have an impact on AD progression and long-term health through two main processes. First, high levels of vitamin C may interact with several types of medications among individuals with preexisting chronic diseases, leading to adverse outcomes [52]. Second, high levels of vitamin C may cause nutritional imbalance that affects the synthesis of other beneficial vitamins and minerals, such as vitamin B12 [52]. Accordingly, low vitamin B12 levels have been linked to brain injury through oxidative stress and tissue damage [52,53]. In the presence of free iron, high levels of vitamin C may act as prooxidants, contributing to oxidative damage or stimulating lipid peroxidation [52,54]. Future studies should consider evaluating the clinical significance of the vitamin C threshold reported in the current study in the development and progression of AD.

The limitations of the current study are as follows. First, only a single measure of serum vitamin C was obtained in this study. With vitamin C being water-soluble and not stored for long periods in the body, the use of a single measure of vitamin C may be reflective of short-term status (1–4 weeks), which may change over time [31]. However, with adverse changes in AD biomarkers occurring about 20 years before the diagnosis of AD [55], it is possible that even short-term exposure to vitamin C may have beneficial effects on AD progression. Second, despite adjusting for several important covariates, it is conceivable that the levels of serum vitamin C may have been influenced by other health conditions and certain medications that were not measured in this study, resulting in the presence of residual confounding. Finally, although a high proportion (98.5%) of deaths in NHANES studies has been reported, the possibility for misclassification of AD-related death by the use of death certificates cannot be entirely ruled out [56].

## 5. Conclusions

This study of a national cohort of community-dwelling elderly adults shows an inverse association between serum vitamin C levels and AD mortality, although beyond concentrations of 2.3 mg/dL, an elevated risk for AD mortality occurs. In light of the increasing incidence of AD in the United States, these findings are of significant clinical and public health importance, as they suggest that serum levels of vitamin C may play a protective role in the development and progression of AD. Confirmation of these findings from prospective studies with repeated measurements of serum vitamin C concentrations is warranted.

## Figures and Tables

**Table 1:** Characteristics of the 4504 community-dwelling elderly adults according to serum levels of vitamin C, NHANES, 1988–1994.

	Serum Vitamin C (mg/dL)			
Characteristics	Tertile 1 (<0.56)	Tertile 2 (0.56–0.98)	Tertile 3 (>0.98)	*p* Values
Age, years	69.5 (7.2)	69.5 (7.10)	70.6 (7.4)	0.017
Sex, male, %	53.7	49.5	29.7	<0.001
Race/ethnicity, %				<0.001
Non-Hispanic White	78.6	83.7	91.4	
Non-Hispanic Black	13.3	7.7	3.4	
Mexican American	3.3	2.2	1.5	
Other	4.8	6.4	3.8	
Some college degree or higher, %	19.2	32.6	32.3	<0.001
Low income, %	34.1	25.3	22.6	<0.001
Marital status, married, %	59.7	63.4	62.7	0.124
Systolic blood pressure, mmHg	140 (19)	138 (19)	138 (19)	0.046
Smoking status, current, %	25.8	10.8	11.5	<0.001
Alcohol intake, past year, %	42.0	47.9	42.3	0.060
Body mass index, kg/m^2^	27.7 (5.3)	27.7 (5.1)	26.1 (4.7)	<0.001
Obese, %	28.0	28.1	19.2	<0.001
Sedentary lifestyle, %	22.5	19.3	15.4	<0.001
Total cholesterol, mg/dL	224 (46)	224 (45)	226 (42)	0.300
Diet quality, Healthy Eating Index	61.3 (13.2)	69.3 (12.7)	72.8 (12.3)	<0.001
Dietary vitamin C intake, mg	65.1 (62.8)	112.7 (94.4)	135.1 (108.4)	<0.001
Vitamin C supplement intake, %	6.2	19.4	42.5	<0.001
Prevalent health conditions, %				
Hypertension	53.6	53.2	48.9	0.079
Diabetes	11.5	11.1	5.9	<0.001
Cancer	18.3	17.7	23.0	0.009
Cardiovascular disease	22.3	16.3	13.7	<0.001

The reported proportions for categorical variables and means (standard deviation) for continuous variables are all survey-weighted values. To convert serum vitamin C from mg/dL to μmol/L, multiply by 56.78.

**Table 2:** The association of serum levels of vitamin C and Alzheimer’s disease-related mortality, NHANES III 1988–2019.

	Serum Vitamin C (mg/dL)			
	Tertile 1 (<0.56)	Tertile 2 (0.56–0.98)	Tertile 3 (>0.98)	*p* Value
Incidence, per 1000	2.9 (2.2–3.7)	2.3 (1.7–3.0)	2.2 (1.6–2.9)	
Model 1	1 (referent)	0.72 (0.36–1.44)	0.51 (0.31–0.84)	0.028
Model 2	1 (referent)	0.66 (0.32–1.33)	0.49 (0.29–0.82)	0.030
Model 3	1 (referent)	0.65 (0.33–1.26)	0.50 (0.30–0.81)	0.024
Model 4	1 (referent)	0.59 (0.29–1.24)	0.44 (0.25–0.77)	0.021

Model 1: Adjusted for age, sex, race/ethnicity, education, poverty–income ratio, and marital status. Model 2: Model 1 plus systolic blood pressure, smoking status, body mass index, abdominal obesity, physical activity, and total cholesterol. Model 3: Model 2 plus prevalent chronic conditions at baseline (diabetes, cardiovascular disease, and cancer). Model 4: Model 3 plus self-reported total dietary vitamin C intake. To convert serum vitamin C from mg/dL to μmol/L, multiply by 56.78.

## Data Availability

Data used for this study are publicly available from the Centers for Disease Control and Prevention at https://www.cdc.gov/nchs/nhanes/ (accessed on 24 May 2024).
